# Retrospective Study of Chronic Coughing in Dogs in a Referral Centre in the UK: 329 Cases (2012–2021)

**DOI:** 10.3390/ani15020254

**Published:** 2025-01-17

**Authors:** Carla Asorey Blazquez, Ico Jolly Frahija, Arran Smith, Rachel Miller, Mayank Seth, Edgar Garcia Manzanilla, Ferran Valls Sanchez

**Affiliations:** 1 DWR Veterinary Specialists, Part of Linnaeus Veterinary Limited’, Station Farm, London Road, Six Mile Bottom, Cambridgeshire CB8 0UH, UK; ico.jolly@gmail.com; 2 Cave Veterinary Specialists, Part of Linnaeus Veterinary Limited’, George’s Farm, NR Wellington, West Buckland, Wellington, Somerset TA21 9LE, UK; 3 Department of Veterinary Medicine, Queen’s Veterinary School Hospital, University of Cambridge, Cambridge, Cambridgeshire CB3 0ES, UK; ajs356@cam.ac.uk; 4 Small Animal Internal Medicine Consultant, Vet Oracle Telemedicine, CVS Referrals, Owen Road, Diss, Norfolk IP22 4ER, UK; rachel.miller@svs.vet; 5 Bsc BVetMed Dip-ECVIM-CA DACVIM FRCVS Clinical Director, Stanstead Veterinary Specialists, Stanstead Courtyard, Parsonage Road, Bishop’s Stortford CM22 6PU, UK; 6 Animal and Grassland Research and Innovation Centre, Teagasc, Moorepark, Cork, Ireland & School of Veterinary Medicine, University College Dublin, Dublin P61 C996, Ireland; egmanzanilla@gmail.com; 7 WeYouVets, Ingrave, Essex CM13 3NU, UK; fvsmedicine@gmail.com

**Keywords:** airway collapse, canine, chronic bronchitis, eosinophilic, foreign body, infectious pneumonia, laryngeal paralysis, neoplasia, respiratory disease, risk factor

## Abstract

Chronic coughing is a common complaint in small animal medicine and it has an extensive differential diagnosis with very different treatment and prognosis. Coughing is considered chronic when it lasts at least 8 weeks. This retrospective study aimed to describe the most common causes of chronic coughing in a population of dogs that presented to a referral hospital in England between January 2012 and December 2021. In addition, we aimed to evaluate if an association with signalment, weight, coughing characteristics, and/or concomitant clinical signs could be identified. A total of 329 dogs met the inclusion criteria. The most frequent diagnoses were airway collapse (102/329, 30.7%), chronic bronchitis (80/329, 24.3%), neoplasia (62/329, 18.8%), and infectious bronchopneumonia (54/329, 16.4%). Other diagnoses were also identified in a lower number of cases. Pomeranian, Chihuahua, and Yorkshire Terrier were over-represented breeds for airway collapse. No predictive factors were identified for chronic bronchitis. Lower body weight (odds ratio [OR] 0.92, 95% confidence interval [CI]: 0.90–0.95), non-productive (OR 14.3 CI 3.44–50) paroxysmal coughing (OR 4.9, 95%, CI: 2.2–11.0), and exercise intolerance (OR 3.3, 95%, CI: 1.7–6.3) increased the odds of airway collapse. Older dogs (OR 1.025, 95%, CI: 1.014–1.036), higher body weight (OR 1.048, 95%, CI: 1.018–1.080), lethargy (OR 5.1, 95%, CI: 1.5–17.7), haemoptysis (OR 8.6, 95%, CI: 1.9–38.4), weight loss (OR 4.0, 95%, CI: 1.1–15.3), and inappetence (OR 6.5, 95%, CI: 1.9–22.1) increased the odds of neoplasia. Productive coughing (OR 3.0, 95%, CI: 1.5–6) and nasal discharge (OR 4.1, 95%, CI: 1.4–11.9) were predictive factors of infectious bronchopneumonia. Younger age (OR 0.96, 95%, CI: 1.061–1.150), higher body weight (OR 1.097, 95%, CI: 1.037–1.161), and haemoptysis (OR 11.8, 95%, CI: 1.8–78.5), increased the odds of airway foreign body. Higher body weight (OR 1.105, 95%, CI: 1.061–1.150), older age (OR 1.041, 95%, CI: 1.022–1.061), and regurgitation were predictive factors of laryngeal paralysis. Epiglottic retroversion was associated with younger age (OR 0.969, 95%, CI: 0.943–0.994), dysphagia (OR 42.5, 95%, CI: 4.7–382.7), and regurgitation (OR 11.6, 95%, CI: 1.2–113.1). All the aforementioned findings are valuable for prioritising differential diagnoses and consequently increasing the efficacy of the diagnostic plan and prioritising tests, which becomes even more important in cases where financial constraints are present. Finally, an appropriate selection of diagnostics tools helps optimise veterinary resources.

## Simple Summary

Chronic coughing is a commonly presenting complaint in dogs, and it can be caused by multiple clinical conditions with very different treatments. The aim of this study was to describe the most common causes of chronic coughing in a population of dogs that presented to a referral hospital in England and to evaluate if there was any association with signalment, weight, coughing characteristics, and concomitant clinical signs. Three-hundred twenty-nine dogs were included. The most common diagnoses were airway collapse, chronic bronchitis, neoplasia, and infectious bronchopneumonia. Lower body weight, non-productive paroxysmal coughing, and exercise intolerance were risk factors for airway collapse, while older age, higher body weight, lethargy, haemoptysis, weight loss, and inappetence were risk factors for neoplasia. Dogs with infectious bronchopneumonia more frequently had productive coughing and nasal discharge. No predictive factors were identified for chronic bronchitis. Pomeranian, Chihuahua, and Yorkshire Terrier were over-represented breeds for airway collapse. Literature assessing chronic coughing in dogs as a general complaint is scarce. Therefore, data that provides guidance for setting a differential diagnosis and diagnostic approach is useful.

## 1. Introduction

Cough is defined as an uncontrolled protective reflex mechanism, with the expulsion of air, with the aim of clearing the airway [1]. Cough can be elicited due to the stimulation of receptors localised in the larynx, trachea, or bronchi, whereas the irritation of smaller bronchi, bronchioles, and alveoli does not elicit coughing [2,3]. In the long term, coughing can lead to mucosal damage and respiratory compromise, which ultimately could have a significant impact on the patient’s quality of life. In small animal practice, cough is a common complaint and indicates an underlying disease process which may not be strictly associated with the airways [4,5].

In human medicine, cough is classified as acute (under 3 weeks duration), subacute (between 3 and 8 weeks duration), or chronic (over 8 weeks duration) [6]. In veterinary medicine, cough is more simply classified as acute or chronic; the latter being a cough of at least 8 weeks duration [3,7,8]. Cough is also often classified as dry, also known as non-productive, or wet, also known as productive. However, from a practical point of view, this classification may be challenging, as secretion may not always be easily perceived by the owners when dogs swallow the sputum. Moreover, an individual with a dry cough may have mucous production, while a dog with a wet cough may not produce sputum.

Historically, cough could also be classified as respiratory or cardiac. However, cardiac cough has always been a topic of debate among veterinarians. Often, cough and cardiac disease occur simultaneously, but are not necessarily linked. Moreover, it is believed that left atrial enlargement is not sufficient to cause cough by itself, but could contribute if there is an underlying respiratory disease [8,9,10].

The causes of chronic coughing in dogs are variable and without a diagnosis and appropriate treatment, the prognosis may be guarded [11]. Reaching a diagnosis in patients with chronic coughing is, therefore, crucial for the outcome of the patient.

Several studies in veterinary medicine have described the most common presentation for specific diseases trying to establish an association between these pathologies and different breeds. An example is toy breeds, who are reported to present frequently with chronic coughing due to tracheal collapse. On the other hand, large breed dogs are rarely affected by this pathology [12,13]. Other examples include large breeds such as Labrador Retrievers, Afghan hounds, Irish Setters, Golden Retrievers, Saint Bernards, and Standard Poodles, who commonly present with acquired laryngeal paralysis [14,15].

Nevertheless, the investigation of chronic coughing can be challenging for veterinarians, particularly in general practice where certain diagnostic tools, such as computed tomography and bronchoscopy, are often not available. Therefore, it seems essential to understand what diagnostic tools are more useful or sufficient for the diagnosis of certain conditions that can lead to chronic coughing. Moreover, on occasions, performing investigations may not be possible due to the owner’s financial situation or the patient’s medical condition, which may limit the investigations performed. In these cases, making a presumptive diagnosis could be the only option and having literature assessing what diagnosis is more likely depending on the breed, dog size, and concomitant clinical signs could be lifesaving.

To the authors’ knowledge, there are no large studies evaluating chronic cough as a general complaint and its association with certain breeds, sizes, and concurrent clinical signs. The main aim of this retrospective study was to evaluate all dogs that presented to a referral centre in the United Kingdom with chronic coughing, describe the most common causes, and evaluate if there were any weight, sex, or breed—disease associations. We also evaluated if certain clinical signs were usually present along with coughing for a particular condition.

## 2. Materials and Methods

The electronic medical records of client-owned dogs presenting with chronic coughing to a referral hospital in the United Kingdom between January 2012 and December 2021, were retrospectively reviewed.

The search was performed using the keywords ‘chronic cough’, ‘chronic coughing’, ‘cough’, and ‘coughing’ in the database of a single referral hospital in the United Kingdom. The referral reports and medical records of all the dogs presenting with chronic coughing over a 9-year period were reviewed.

The dogs were included if they were presented with coughing of at least 8 weeks duration according to the carer’s report, had complete medical records, and a definitive diagnosis had been achieved, or a clinical diagnosis was established based on the clinician’s judgement. Complete medical records included signalment, body weight, length of the coughing, description of the coughing, and diagnostic investigations performed on each case. The investigations performed for each patient were at the clinician’s discretion. The results of these diagnostic investigations were reviewed by C.A.B and F.V.S to determine whether they supported the diagnoses recorded for every individual. The results of each test and laboratory result for every dog were not included in the manuscript to maintain readability and fluidity. If the dogs were diagnosed with more than one condition that could be responsible for the coughing, both were recorded.

Signalment (breed, age, sex, and neuter status) and body weight were included for all the cases. The age was recorded in months and the body weight in kilograms. The duration of the coughing from the onset of the clinical signs up to the moment of presentation at the referral centre was recorded using weeks as a unit. The type of cough was documented as productive or non-productive. The classification of the cough was made based on the active witnessing of the coughing, description on medical records of the clinician in charge of the case, the referring veterinarians or the carer’s perception, and subsequent description. Paroxysmal coughing episodes were also documented in the study and recorded as ‘present’ or ‘not present’ for each case. Paroxysmal coughing is defined in human medicine as recurrent prolonged coughing episodes with an inability to breathe during spells [16], but in veterinary medicine, a standardised definition is not reported. However, the term paroxysmal is often used to define the sudden onset of a clinical sign. In this manuscript, paroxysmal coughing refers to a sudden onset and/or violent coughing.

Concomitant clinical signs present at the time of the initial consultation at both the referral centre and the referring veterinarian practice were logged. The treatments that each patient received at any point, either prior to presentation, during investigations, or after diagnosis was achieved, were not documented.

Different groups were made based on the diagnosis (underlying cause of the cough) once all the results were reviewed for each patient. On occasions, a diagnosis could include various forms of disease if they had common features to simplify the results. An example of this was eosinophilic lung disease which was the term elected to describe eosinophilic bronchitis, eosinophilic granuloma, and eosinophilic bronchopneumopathy. Given that often, dogs with tracheal collapse have concurrent bronchial collapse and vice versa, the term ‘airway collapse’ was used for all the dogs diagnosed with tracheal or bronchial collapse, and/or bronchomalacia to simplify the results. In this study, the diagnosis of airway collapse was made if either tracheal or bronchial collapse, or bronchomalacia were identified on fluoroscopy, thoracic radiography, computed tomography (CT), and/or bronchoscopy. If a dog had a cervical or intrathoracic mass causing a degree of airway compression (tracheal or bronchial), the final diagnosis recorded was the mass (e.g., neoplastic process) rather than the airway collapse. If a mass was identified in a dog without causing airway compression, and bronchoscopy identified concurrent tracheal or bronchial collapse, or bronchomalacia, both diagnoses were recorded for that patient. The dogs with laryngeal collapse were classified as a separate group as the pathophysiology and clinical presentation of laryngeal collapse differs from tracheal and bronchial collapse. A diagnosis of infectious disease as the cause of the coughing was made in the dogs with bacterial, parasitic, viral, or fungal bronchopneumonia, confirmed on culture or PCR for a specific infectious agent. The dogs were diagnosed with neoplasia if cytology or histopathology confirmed a neoplastic process (either primary or metastatic), or if imaging alone showed one or multiple intrathoracic nodules compatible with a neoplastic process based on appearance and distribution, but sampling was not possible due to location, or if a primary neoplastic process was identified in other cavity/organ, confirmed to be neoplasia, along with multiple pulmonary nodules, compatible with metastatic disease. Laryngeal paralysis and epiglottic retroversion were diagnosed based on a direct assessment of the laryngeal movement on voluntary respiration (inspiration and expiration) under sedation. When the laryngeal hemiplegia of one or both arytenoid cartilages was identified, a diagnosis of laryngeal paralysis was made. The dogs were diagnosed with chronic bronchitis if a bronchoalveolar lavage (BAL) cytology confirmed neutrophilic and/or macrophagic inflammation and/or goblet cell hyperplasia without the identification of another significant underlying cause that could justify the chronic coughing. A diagnosis of chronic bronchitis could also be made when there was a concurrent diagnosis that could cause or contribute to the coughing, along with marked bronchial changes (e.g., severe bronchial thickening or bronchiectasis) visualised on bronchoscopy, which could not be explained by the other pathology identified. A diagnosis of an airway foreign body was made if a foreign body was identified during the direct examination of the larynx; CT of the head, neck, or thorax; or bronchoscopy.

The CTs were performed using a 16-slide multidetector-row unit (MX-16 Philips, Healthcare). Thoracic radiographs were taken using the Agfa CR35X model, and thoracic ultrasounds were performed using a Philips iU22 model. A variety of endoscope sizes and brands were used during the 10-year period.

Dogs were excluded if the cause of the coughing was concluded only in the light of the signalment and clinical signs, a clinical definitive diagnosis was not stated, or the diagnostics tests leading to the final clinical diagnosis in the report were considered equivocal by C.A.B and F.V.S upon revision of the data. Dogs were also excluded if the length of the coughing was not known or if the medical records were incomplete.

### Data Analysis

All the analyses were carried out using SAS 9.4 (SAS Institute, Cary, NC, USA) and R 4.4.1 (R Core Team 2021, Vienna, Austria). An individual dog was considered as the experimental unit. The alpha level for the determination of significance was 0.05 and trends are reported for alpha 0.10. Multiple comparisons were adjusted with Bonferroni’s correction when required. All the continuous variables were checked for normality using graphical methods and Kolmogorov–Smirnov tests. The dependent or outcome variables were the presence of each of the diagnostics (binary). The independent or predictor variables included signalment, coughing characteristics, diagnostic tests performed, and clinical signs. The associations between diagnosis and the diagnostic test were calculated for each pair of outcome and predictor variables by univariate analysis using Chi-square or Fisher’s exact test. The co-occurrence of diagnostics was studied using Fisher’s exact test. The associations between breed, chronic coughing, and diagnosis were studied by considering the total number of patients of each breed during the same period of time using the Chi-square test. A multivariable model was developed for each diagnosis as a binary outcome variable including all the clinical record data (age, weight, sex, and breed) and all the clinical signs as predictors. The relation between each pair of outcome and predictor variable was calculated by univariate analysis using Chi-square or Fisher’s exact test in the case of categorical variables and by the Wilcoxon–Mann–Whitney test for continuous variables. Multivariable analysis of each diagnosis was carried out using logistic regression. Variables were selected for inclusion in the regression analysis if the *p*-value calculated for their association with the outcome variable in the univariable analysis was ≤0.25 and the variable was considered to be biologically significant. In the case of co-linearity among predictors, the one to be included was selected based on its association with the outcome in the univariate analysis. The model was fitted by the manual stepwise procedure.

## 3. Results

### 3.1. Descriptive Study of the Population

The study population included 329 dogs. Table 1 shows a summary of the signalment data at presentation. The dogs had a mean age of 101 months (8.4 years; 5-193 months) and an average body weight of 17.2 kg (1.3–51.8 kg). The population was composed of 45% females (148/329) and 55% (181/329) males, of which 24% were entire dogs (31/329, 9.4% entire females and 48/329, 14.6% entire males) and 76% were neutered dogs (117/329, 35.6% neutered females and 133/329, 40.4% neutered males). Most dogs were purebred (277/329, 84.2%) and the most common breeds were Labrador Retriever (36/329, 10.9%), Yorkshire Terrier (23/329, 7.0%), Cocker Spaniel (18/329, 5.5%), Springer Spaniel (17/329, 5.2%), Pomeranian (10/329, 3.0%), Chihuahua (10/329, 3.0%), Cavalier King Charles Spaniel (9/329, 2.7%), Labradoodle (8/329, 2.4%), and Jack Russell Terrier (8/329, 2.4%).

The mean duration of the coughing at presentation was 35.5 weeks (8.9 months). Non-productive coughing was reported in most dogs (280/329, 85.1%) and productive coughing in the remaining 14.9% (49/329). Paroxysmal coughing was reported in 16.1% (53/329) dogs. The most common concomitant clinical sign was exercise intolerance affecting 22.5% (74/329) of the dogs. Other clinical signs reported were lethargy (32/329, 9.7%), dyspnoea (29/329, 8.8%), tachypnoea (28/329, 8.5%), sneezing (27/329, 8.2%), inappetence (19/329, 5.7%), nasal discharge (16/329, 4.9%), weight loss (14/329, 4.3%), haemoptysis (13/329, 4.0%), vomiting (12/329, 3.6%), dysphagia (10/329, 3.0%), fever (9/329, 2.7%), stridor (8/329, 2.4%), retching (8/329, 2.4%), stertor (7/329, 2.1%), bark change (6/329, 1.8%), regurgitation (6/329, 1.8%), positive tracheal pinch (5/329, 1.5%), syncopal episodes (5/329, 1.5%), and cyanosis (4/329, 1.2%).

All the dogs underwent investigations to achieve a diagnosis. The following tests were carried out in most dogs (equal or more than 50%): haematology (282/329, 85.7%), biochemistry (268/329, 81.5%), thoracic radiography (239/329, 72.6%), upper airway examination (215/329, 65.4%), bronchoscopy (247/329, 75.1%), and cytology of the bronchoalveolar lavage (238/329, 72.3%). 

From the 329 dogs included in the study, 102 were diagnosed with airway collapse, accounting for tracheal and/or bronchial collapse (102/329, 30.7%); chronic bronchitis was identified in 80 dogs (80/329, 24.3%); neoplasia in 62 (62/329, 18.8%); infectious bronchopneumonia (including bacterial, parasitic, viral, and fungal) was diagnosed in 54 dogs (54/329, 16.4%); 38 dogs suffered eosinophilic lung disease (38/329, 11.6%); 29 were diagnosed with laryngeal paralysis (29/329, 8.8%); and an airway foreign body (laryngeal, tracheal, bronchial, parenchymal, or pleural) was identified in 15 dogs (15/329, 4.6%). The following diagnoses were identified in a smaller number of cases, including epiglottic retroversion (6/329, 1.8%), aspiration pneumonia (4/329 1.2%), pulmonary fibrosis (4/329 1.2%), laryngitis (3/329, 0.9%), and other (13/329, 4.0%).

A total of 69 dogs (69/329, 20.9%) were diagnosed with more than one condition at presentation.

Table 2 shows the co-occurrence of diagnoses. Significant associations (*p* < 0.05) were found between airway collapse and infectious bronchopneumonia (15/102, 14.7%), chronic bronchitis and infectious bronchopneumonia (14/80, 17.5%), eosinophilic lung disease and infectious bronchopneumonia (4/38, 10.5%), airway collapse and laryngeal paralysis (6/102, 5.9%), and chronic bronchitis and laryngeal paralysis (8/80, 10%).

### 3.2. Association Between Signalment, Type of Coughing and Diagnosis

Table 3 shows the univariate analysis of the continuous variables (duration of the coughing, age, and body weight) for all the cases and for each diagnosis. The duration of coughing at presentation was longer for the dogs with airway collapse (42.8 weeks; *p* = 0.007) and epiglottic retroversion (93.3 weeks; *p* = 0.026) and shorter in neoplasia (25.9 weeks; *p* = 0.004) and airway foreign body (16.9 weeks; *p* = 0.028) compared to the mean coughing duration for all the diagnoses.

The mean age of presentation for the investigations of chronic coughing was 101 months (8.4 years). The dogs diagnosed with neoplasia and laryngeal paralysis were overall older at presentation (127.7 and 136.1 months, respectively; *p* < 0.001). The dogs were younger for infectious bronchopneumonia (88.7 months; *p* = 0.030), eosinophilic lung disease (63.3 months; *p* < 0.001), airway foreign body (47.7 months; *p* < 0.001), and epiglottic retroversion (47.8 months; *p* = 0.009) compared to the overall value for all the diagnoses.

In regard to the weight, the mean weight when accounting for all the dogs was 17.2 kg. The dogs had higher body weight at presentation for chronic bronchitis (19 kg; *p* = 0.039), neoplasia (21.6 kg; *p* < 0.001), laryngeal paralysis (28.3 kg; *p* < 0.001), and airway foreign body (24.9 kg; *p* = 0.005). On the contrary, the dogs diagnosed with airway collapse had lower body weight (10.8 kg; *p* < 0.001), as well as the dogs diagnosed with epiglottic retroversion (6.9 kg; *p* = 0.006) compared to the overall mean body weight for all the diagnoses.

No associations were identified between the frequencies of sex or neutering status with any of the diagnoses. Associations between breed and chronic coughing are shown in Table 4. The overall prevalence of chronic coughing was 0.79% for the period studied. The breeds showing higher prevalences of chronic coughing were Pomeranian (5.1%), Irish Terrier (3.8%), Yorkshire Terrier (3.1%), Toy Poodle (2.6%), Shetland Sheepdog (2.6%), Whippet (1.7%), and Labradoodle (1.4%).

Regarding coughing characteristics, paroxysmal episodes were positively associated with airway collapse (*p* < 0.001) and negatively associated with neoplasia (*p* = 0.007). Productive coughing was positively associated with infection (*p* = 0.026), eosinophilic lung disease (*p* = 0.049), and foreign body (*p* = 0.014), and negatively associated with airway collapse (*p* < 0.001).

### 3.3. Association Between Diagnostic Tests and Diagnosis

In regard to the diagnostic tests and procedures performed to investigate the coughing, Table 5 summarises the tests carried out more frequently for each diagnosis, and which tests were less frequently performed for certain diagnoses. The complete analysis can be found in Supplementary Table S1.

Airway collapse was associated with bronchoscopy, performed in 93% of the dogs with this diagnosis (*p* < 0.001), as well as BAL (84%, *p* = 0.001), thoracic radiographs (81%, *p* = 0.022), culture of the BAL (79%, *p* = 0.005), upper airway assessment (78%, *p* = 0.001), fluoroscopy (12.9%, *p* < 0.001), and echocardiography (29.7%, *p* < 0.001).

All the dogs with chronic bronchitis had a BAL (100%, *p* < 0.001) and 98% of the dogs with this diagnosis underwent a bronchoscopy (98%, *p* < 0.001). Culture of the BAL was performed in 92% of the cases with chronic bronchitis (*p* < 0.001) as well as Polymerase chain reactions (PCR) for infectious diseases (45%, *p* < 0.001), particularly *Mycoplasma cynos* PCR (39%, *p* < 0.001); *Angiostrongylus vasorum* IDEXX Angio Detect Test^TM^) (47%, *p* < 0.001); faecal analysis (18% *p* = 0.001); and upper airway assessment (77%, *p* = 0.010).

The dogs diagnosed with a neoplastic process frequently underwent CT (71%, *p* < 0.001), thoracic ultrasound (32%, *p* < 0.001), fine needle aspirates (FNA) (62%, *p* < 0.001), biopsies for histopathology (33%, *p* < 0.001), and coagulation times (12%, *p* = 0.044).

Infectious bronchopneumonia was diagnosed in most cases by performing bronchoscopy (88%, *p* = 0.010), BAL (88%, *p* = 0.002), culture (94%, *p* < 0.001), and PCRs for infectious diseases (44%, *p* < 0.001), in particular *Mycoplasma cynos* (38% *p* < 0.001) and *Bordetella* (13% *p* < 0.001).

Bronchoalveolar lavage was the diagnostic procedure more frequently performed in dogs with eosinophilic lung disease. In total, 100% of the cases diagnosed with this pathology underwent a BAL (100%, *p* < 0.001), followed in frequency by bronchoscopy (97%, *p* < 0.001), culture of the BAL (97%, *p* < 0.001), upper airway assessment (81% *p* = 0.029), *Angiostrongylus vasorum* IDEXX Angio Detect Test^TM^, (57%, *p* < 0.001), faecal analysis (21%, *p* = 0.010), *Angiostrongylus vasorum* PCR (10.5%, *p* = 0.002), and *Crenosoma vulpis* PCR (7%, *p* = 0.005).

For laryngeal paralysis, the diagnostic test more frequently performed was upper airway assessment in 100% of the cases (*p* < 0.001), followed by thoracic radiographs (89%, *p* = 0.030).

Lastly, the diagnostic test more frequently performed for the diagnosis of epiglottic retroversion was upper airway assessment in 100% of the cases (*p* = 0.096).

### 3.4. Association Between Clinical Signs and Diagnosis

Table 6 shows the associations between each diagnosis and sex, coughing characteristics, and clinical signs in a univariate analysis and Table 7 shows the results for the multivariable analysis including signalment, coughing characteristics, and clinical signs.

In the univariate analysis, a diagnosis of airway collapse was associated with longer duration of coughing, paroxysmal coughing, non-productive coughing, lower body weight, exercise intolerance, dyspnoea, and the absence of nasal discharge, haemoptysis, or vomiting. In the multivariable analysis, the odds of chronic coughing being caused by airway collapse were 4.9 and 3.2 times higher in the dogs with paroxysmal coughing episodes and exercise intolerance, respectively (*p* < 0.001) and 14.3 times lower in the dogs with productive coughing (*p* < 0.001). Body weight was negatively associated with airway collapse (*p* < 0.001). The dogs weighing less than 10 kg had increased odds (2.5 times) of being diagnosed with airway collapse.

In the univariate analysis, a diagnosis of chronic bronchitis was associated with higher body weight and the absence of weight loss or haemoptysis. However, in the multivariable analysis, no predictors were significant for chronic bronchitis.

In the univariate analysis, a diagnosis of neoplasia was associated with a shorter duration of coughing, older age, higher body weight, weight loss, inappetence, haemoptysis and vomiting, and lowest frequency of exercise intolerance, paroxysmal coughing, or sneezing.

In the multivariable analysis, the odds of chronic coughing being caused by a neoplastic process were 5.1 and 4.0 times higher for the dogs presenting with lethargy (*p* = 0.010) or weight loss (*p* = 0.041), respectively, 8.6 times higher in the dogs with haemoptysis (*p* = 0.005), 6.5 times higher with inappetence (*p* = 0.003), and 88% lower when the dogs presented with exercise intolerance (*p* < 0.001). The odds of neoplasia also increased by 1.024 and 1.048 with an additional month of age (*p* < 0.001) and kg of body weight (*p* = 0.002), respectively. For an increase of 1 year of age or 10 kg of body weight, the odds of presenting with chronic coughing due to a neoplastic process were 1.35 and 1.6 times higher, respectively.

In the univariate analysis, a diagnosis of infection was associated with younger age, productive coughing, and nasal discharge. In the multivariable analysis, the odds of chronic coughing being caused by an infection were 3.0 times higher when the dogs presented with productive coughing (*p* = 0.002) and 4.1 times higher in the dogs with nasal discharge (*p* = 0.009).

In the univariate analysis, a diagnosis of eosinophilic lung disease was associated with younger age, productive coughing, sneezing, and absence of lethargy. In the multivariable analysis, the odds of chronic coughing being caused by eosinophilic lung disease were 3.2 times higher when the dogs presented with sneezing (*p* = 0.022) and 22.5% lower for an increase of 1 year of age (*p* < 0.001).

In the univariate analysis, a diagnosis of laryngeal paralysis was associated with older age, higher body weight, and a change in bark. However, in the multivariable analysis, the odds of chronic coughing being caused by laryngeal paralysis were 48.5 times higher when the dog presented regurgitation (*p* = 0.002), 2.7 times higher for an increase of 10 kg in body weight (*p* < 0.001) and 3.3 times higher for an increase of 1 year in age (*p* < 0.001).

In the univariate analysis, a diagnosis of foreign body was associated with shorter duration of coughing, younger age, higher body weight, and productive coughing. In the multivariable analysis, the odds of chronic coughing being caused by a foreign body were 11.8 times more likely if the dog presented haemoptysis (*p* = 0.010), 2.5 times higher for an increase of 10 kg in body weight (*p* = 0.001), and 37.5% lower for an increase in each year of age (*p* < 0.001).

Finally, in the univariate analysis, a diagnosis of epiglottic retroversion was associated with a longer duration of coughing, younger age, lower body weight, dysphagia, and regurgitation. In the multivariable analysis, the odds of chronic coughing being caused by epiglottic retroversion were 42.5 and 11.6 times higher for the dogs presenting dysphagia (*p* < 0.001) or regurgitation (*p* = 0.035), respectively, and 31.9% lower for an increase in each year of age (*p* = 0.017).

## 4. Discussion

Chronic cough is frequently encountered in small animal medicine and different conditions can be responsible for it. Literature assessing chronic coughing in dogs as a general complaint is scarce. Consequently, data that could provide guidance for setting a differential diagnosis and diagnostic approach would be useful. This study aimed to identify the most common causes of chronic coughing in dogs and if there was any association between specific diseases and signalment, clinical signs, and coughing characteristics.

In this study, airway collapse and chronic bronchitis were the most common causes of chronic coughing, followed by neoplasia and infectious bronchopneumonia. This correlates with a previous prospective study, which evaluated demographic and historical findings in 115 dogs with chronic coughing, and identified chronic bronchitis and airway collapse as the most common diagnoses [17].

### 4.1. Association Between Body Weight and Coughing

Certain diagnoses were more or less frequent depending on the body weight. The likelihood of airway collapse was reduced in large breed dogs; however, it is important to bear in mind that in this study, this group included tracheal and bronchial collapse. On the contrary, dogs weighing less than 10 kg had increased odds (2.5 times) of being diagnosed with airway collapse. In contrast, increased body weight was associated with a higher risk of being diagnosed with a neoplastic process. A 10 kg increase in body weight increased by 1.6 times the odds of presenting with chronic coughing due to neoplasia. Similarly, the dogs with higher body weight also appeared to have a higher risk of laryngeal paralysis or an airway foreign body. The latter could be due to different activity patterns. For example, it could be speculated that larger dogs are often more playful and frequently live in rural areas or larger houses with access to a garden, whilst smaller dogs frequently live in urban areas with less exposure to common airway foreign bodies.

### 4.2. Association Between Signalment and Coughing

The mean age at presentation was 8.4 years, which is also similar to previous studies, with a reported average age of 9.4 [17] and 8 years old [18] for dogs presenting with chronic coughing.

The dogs diagnosed with laryngeal paralysis were overall older at presentation than the average in this population. These results were expected given that most cases of laryngeal paralysis in dogs are acquired and suspected to be due to a generalised peripheral polyneuropathy, more commonly referred to as geriatric onset laryngeal paralysis polyneuropathy; typical of old, large breed dogs; and considered a degenerative process [15,19]. The same occurred with the dogs diagnosed with a neoplastic process who were significantly older at presentation in our study. For an increase of 1 year of age, the odds of presenting with chronic coughing due to a neoplastic process and laryngeal paralysis were 1.35 and 3.3 times higher, respectively.

Older age decreased the risk of airway foreign body and eosinophilic lung disease. The former can be explained due to behavioural patterns at different ages. Eosinophilic lung disease could be most commonly encountered in younger dogs due to its pathophysiology. A type II hypersensitivity response is hypothesised as a cause of eosinophilic lung disease in the literature, and it could be speculated that exposure to an allergen is more likely to occur earlier in life with consequent clinical signs.

In our study, age was not a risk factor for dogs for airway collapse, which was interesting, since it is more frequently reported in middle-aged to older dogs [13].

Most dogs were purebred (84.2%). Pomeranians, Yorkshire Terriers, Irish Terriers, Shetland Sheepdogs, Toy Poodles, Whippets, Labradoodles, and Chihuahua showed higher prevalence of chronic coughing compared to the overall population of dogs presented within the study period (January 2012 and December 2021). This correlates with previous studies that identified Pomeranians, Chihuahuas, Yorkshire Terriers, Toy Poodles, and Shetland Sheepdogs as over-represented breeds for coughing [17]. We also identified an association between Pomeranians, Yorkshire Terriers, and Chihuahuas and airway collapse, a finding that has been reported in previous studies as well [12,20,21].

The aetiology of tracheal and bronchial collapse is poorly understood and is suspected to be multifactorial [12,21]. Nevertheless, the frequency of airway collapse in small breed dogs and the associations identified for certain breeds could lead to speculation about a genetic component to the disease. The investigation of a possible genetic component could allow the improvement of breeding practices to reduce disease prevalence.

Cocker Spaniels with chronic coughing have also been over-represented in previous studies [17], and breed predisposition to bronchiectasis has also been reported [22]. Interestingly, this breed predisposition to chronic lower airway inflammation and bronchiectasis was not identified in our study.

No other breed associations were identified. Five dogs presented with chronic coughing and epiglottic retroversion in our study, of which two were Chihuahuas and one was a Chihuahua cross. Unfortunately, the numbers were too low to identify any association with this breed; however, small breed dogs appeared to be over-represented for epiglottic retroversion in the literature [23]. Further studies are warranted to investigate any breed predisposition to this condition.

### 4.3. Duration and Type of Coughing

The time between the reported onset of the cough and presentation to the referral centre varied in our study. The mean duration of coughing was 35.5 weeks (almost 9 months); however, the dogs with an airway foreign body or a neoplastic process presented for investigations sooner than the dogs with other conditions. In particular, the dogs diagnosed with an airway foreign body had a mean coughing duration of 4 months. It is likely that the dogs with an airway foreign body developed a sudden cough, and often became unwell if there was concurrent pyrexia, which probably prompted owners to take their dogs to be assessed by a veterinarian shortly after the onset of the clinical signs. This differs from other conditions, such as airway collapse, in which cough develops progressively over a long period of time and owners may not suspect that there is a significant underlying process until the cough becomes frequent and more violent. Nevertheless, these results may not be completely representative of what accounts in reality, as we recorded the length of coughing from the onset of the clinical signs up to the consultation at the referral centre. Therefore, most dogs had already been assessed by a general practitioner and had treatments that may have alleviated the clinical signs, leading to a delay in referral. An example would be dogs with an airway foreign body that develop a secondary bacterial infection. These dogs likely had antibiotic therapy prior to referral, which could have led to temporary improvement and were referred when clinical signs relapsed after the discontinuation of the antibiotics.

Tracheal narrowing results from the weakening of the tracheal cartilage, which ultimately results in a persistent dry, paroxysmal “goose-honk” cough, tracheal sensitivity, and varying degrees of respiratory difficulty [24]. Paroxysmal coughing was reported in 64% of the dogs with airway collapse in our study and the odds of chronic coughing being caused by airway collapse were 4.9 times higher for the dogs presenting with paroxysmal coughing.

We also identified that the dogs with non-productive coughing were 14 times more likely to have airway collapse. In our study, airway collapse was also associated with exercise intolerance and dyspnoea. These clinical signs have been reported before in dogs with airway collapse [25]. The absence of haemoptysis, vomiting, and/or nasal discharge was associated with a decreased risk of having airway collapse. On the contrary, neoplasia was associated with haemoptysis. The risk of having chronic coughing due to a neoplastic process increased by 8.6 times in the dogs with haemoptysis. It was also associated with lethargy, weight loss, and decreased appetite. However, the dogs with exercise intolerance were less likely to have a neoplastic process causing chronic coughing in our study. Interestingly, a previous study that evaluated the underlying causes of haemoptysis in 36 dogs identified bacterial pneumonia as the most common cause [26]. In our study, no dog with bacterial bronchopneumonia had haemoptysis reported and therefore, such an association was not identified. However, we did identify an association between haemoptysis and airway foreign body. This association appears logical given the expected mechanical trauma caused by a foreign material within the airway and the violent coughing episodes that may result from trying to expel the foreign body. In addition, although there was not a significant association, three patients in our study had haemoptysis and were diagnosed with eosinophilic lung disease. This has been previously reported in humans as a rare presentation [27], although to our knowledge this has not been reported in dogs. The pathophysiology is not well understood. In human medicine, it has been proposed that the eosinophils invade pulmonary basement membranes and vasculature, directly causing damage to the alveoli in the process [28,29].

In regard to infectious bronchopneumonia, we identified an association with productive coughing and nasal discharge, which increased the odds by 3.0 and 4.1 times, respectively.

The dogs with eosinophilic lung disease were associated with sneezing on the multivariable analysis. The odds of chronic coughing being caused by eosinophilic lung disease were 4.1 times higher when the dog presented sneezing. This was an interesting result, as sneezing is not reported frequently with eosinophilic lung disease. On the other hand, nasal discharge has been reported as a common clinical sign of this pathology. However, an association between nasal discharge and eosinophilic lung disease was not identified in our study. A recent study that evaluated eosinophilic bronchitis, eosinophilic granuloma, and eosinophilic bronchopneumopathy in 75 dogs identified nasal discharge in 28% of the dogs [30], and another study that evaluated eosinophilic bronchopneumopathy in dogs identified nasal discharge in 52% of the dogs [31]. It is possible that in our cases, sneezing was a consequence of a small amount of nasal discharge, perhaps not yet visible or considered not significant by their owners and therefore not reported. Nevertheless, this is a speculation and due to the retrospective nature of our study, this could not be more thoroughly assessed.

### 4.4. Association Between Concomitant Clinical Signs and Diagnoses

Dogs with laryngeal paralysis often presented with voice changes and regurgitation. This could be because most cases of laryngeal paralysis are old and have suspected geriatric onset laryngeal paralysis polyneuropathy. The degeneration of the recurrent laryngeal nerve can result not only in laryngeal paralysis with subsequent upper respiratory obstruction but also in oesophageal dysmotility leading to regurgitation [32]. Interestingly, stridor was not a significant predictor for laryngeal paralysis in our study. The reason for this is not clear, since stridor is often one of the most common clinical signs documented in dogs with laryngeal paralysis. We believe that it is possible that owners missed the stridor if it was not frequent. It is also possible that the clinician in charge of these cases in some instances failed to record this clinical sign. However, although dogs with laryngeal paralysis can often present with chronic coughing, many will instead present with stridor as the main clinical sign and without coughing. On other occasions, dogs may present in an acute manner, and therefore, due to our inclusion criteria, those cases would not have been included.

Lastly, in regard to epiglottic retroversion, dysphagia and regurgitation were found to be associated. The odds of chronic coughing being caused by epiglottic retroversion were 42.5 and 11.6 times higher for the dogs presenting dysphagia or regurgitation, respectively. These factors could appear similar to those associated with laryngeal paralysis; however, epiglottic retroversion was also associated with younger age and not necessarily higher body weight. Interestingly, in the literature, stridor and dyspnoea were the most common clinical signs in dogs with epiglottic retroversion [23], while in our study, dyspnoea was only identified in two dogs and stridor was not reported. Nevertheless, it is possible that the owners failed to identify or report stridor and due to the retrospective nature of our study, this could not be further evaluated. Moreover, the small number of cases with epiglottic retroversion in our study does not allow us to make strong conclusions.

### 4.5. Diagnostic Investigations

Different diagnostic investigations were performed in this study depending on the clinician’s judgement for each case. For the diagnosis of airway collapse, 93% of the dogs underwent bronchoscopy and 84% of the dogs had a BAL performed. The investigations were carried out in a referral hospital, which could, therefore, not mirror the investigations performed routinely in general practice. Bronchoscopy is a useful diagnostic tool for the diagnosis of airway collapse, as it allows the direct visualisation of the airways during respiration; however, it is not the only way to reach this diagnosis. Of the 102 dogs with airway collapse diagnosed in this study, 33 dogs had more than one diagnosis, with 15 dogs having concurrent infectious bronchopneumonia, which was statistically significant. Therefore, despite a confirmed diagnosis of airway collapse by the visualisation of the airways, the collection of a BAL would appear beneficial to address other potential causes contributing to chronic coughing.

Echocardiography was not performed in a large number of cases with airway collapse (30%) but was statistically significant. Given that the dogs with airway collapse were overall small breeds, with lower body weight, and also predisposed to myxomatous mitral valve disease, it is possible that echocardiography was performed in these cases to assess potential risks towards general anaesthesia, required for more thorough investigations and invasive procedures, such as bronchoscopy and the collection of a BAL, particularly in cases that had a heart murmur identified on physical examination. Nevertheless, cardiac disease causing coughing by itself has always been a controversial topic. Some clinicians are inclined to think that left atrial enlargement and congestive heart failure can cause chronic coughing by itself, while others believe that there is an underlying cause for the coughing, which can be aggravated with certain cardiac conditions. Given the retrospective nature of our study, it was not possible the understand the underlying reasoning for these dogs to undergo cardiac assessment. Cases referred for the investigations of chronic coughing with advanced cardiac disease often did not meet the inclusion criteria in our study, as the dogs were often not stable enough to undergo procedures to exclude a concurrent primary respiratory pathology. Therefore, it was not possible to know if atrial enlargement and/or pulmonary oedema were potential causative or contributing factors to the coughing in these dogs. In addition, when the data of these cases was reviewed, the authors noted that the description of the onset and duration of coughing was often vague, and although some owners reported an improvement of the coughing after introducing diuretic therapy, in many cases a degree of coughing persisted despite the resolution of the congestive heart failure.

For the confirmation of lower airway inflammation, leading to a diagnosis of chronic bronchitis, a BAL was required and performed in all the cases with this diagnosis. We could think that bronchoscopy may not be necessary, as the most important would be the BAL for the confirmation of lower airway inflammation. Bronchoscopy was performed in the majority of cases in our study, most likely to assess the airways in detail, allow excluding other potential causes of chronic coughing, and allow endoscopic guided BAL (for example from a particularly affected area). Nevertheless, it is important to mention that these cases underwent investigations in a referral hospital where bronchoscopy was widely available and routinely performed, and therefore, this may not be representative of the investigations routinely performed in general practice.

In regard to a neoplastic process in our study, CT was the imaging modality of choice in most cases to locate the disease and plan ultrasound-guided sampling (FNAs and biopsies) and/or surgical planning. However, a neoplastic process can often be identified on thoracic radiographs.

All the dogs diagnosed with eosinophilic lung disease had a BAL performed for the confirmation of an increased proportion of eosinophils within the airways. This procedure was accompanied by a bronchoscopy in 97% of the dogs with this diagnosis. Given that an increase in the eosinophilic component within the airways was identified in these dogs, a parasitic infection was also considered a potential differential diagnosis and therefore, these dogs were frequently tested for *Angiostrongylus vasorum* (IDEXX Angio Detect Test^TM^, PCR, and faecal analysis), as well as *Crenosoma vulpis* PCR and culture of the BAL.

Lastly, for the diagnosis of laryngeal paralysis and epiglottic retroversion, the direct visualisation of the larynx during voluntary respiration under sedation was performed in all the cases in our study. Endoscopy of the larynx can be performed instead as well as the conscious ultrasound of the larynx, but direct visualisation is usually enough to achieve a diagnosis and it is an easy, fast, and economic procedure.

### 4.6. Limitations

There are inherent limitations of this study due to its retrospective nature. The cases were included if the diagnostic procedures performed were considered enough to reach a definitive diagnosis. In addition, the definitive diagnosis was stated based on the results and clinicians’ judgement, and some diagnoses were made by the exclusion of other disease processes. The determination of the duration of the cough, type of cough and concomitant clinical signs was based on the owner’s perception and clinicians’ judgement. In particular, the duration of the cough was recorded based on the owner’s memory, which could have led to inaccuracies, particularly when the owner is uncertain about an exact timeline, and ultimately could have had an impact on the inclusion criteria. Nevertheless, some minor imprecisions in the owner’s memory are expected and were thought to be small enough that the overall outcome of the study would not be significantly affected.

A causal link between diagnostic findings and coughing cannot be concluded in a definitive manner, and in some cases in particular, reasonable doubt from an evidence point of view could be argued. In addition, in cases with more than one diagnosis, it is possible that only one was responsible for the chronic coughing. The presence of concomitant processes occurs sometimes in small animal medicine, and the clinical significance of each diagnosis and its contribution to the clinical signs is difficult or impossible to conclude. This difficulty is even bigger when this relation is assessed in a retrospective manner and based on reports. An example of this is cases diagnosed with a pathology capable of causing chronic coughing, such as airway collapse or chronic bronchitis, with concurrent bacterial bronchopneumonia. The clinical significance of bacterial bronchopneumonia in these cases is uncertain, and perhaps it is a secondary infection that aggravates the already pre-existing coughing.

When the data were retrospectively reviewed, all the causes of chronic coughing identified in this population of dogs were grouped based on the final diagnoses. To simplify the understanding of the study, the dogs with tracheal collapse were grouped along with the dogs with bronchial collapse and bronchomalacia, under the term ‘airway collapse’, which could have had an impact on the results. Bronchomalacia and bronchial collapse are inconsistently defined terms in dogs. However, regional to diffuse dynamic airway collapse of segmental and/or subsegmental bronchi with associated clinical signs due to airflow limitation, has been proposed as a definition for canine bronchomalacia [11]. In the recent literature, the term ‘bronchial collapse’ in dogs was defined as the collapse of larger airways, proximal to segmental bronchi, further categorised by location, type of collapse, and grade (I-III) [33]. Overall, the underlying cause of cartilage softening is not fully understood but may be associated with congenital conditions, extrinsic compression, chronic inflammation, or alterations in elastic fibres [34,35,36]. We could, then, think that our study may have missed risk factors that could have been identified if the conditions were grouped independently, particularly for bronchomalacia, bronchial collapse, and tracheal collapse. For example, in the literature, bronchial collapse or bronchomalacia can commonly be seen in medium to large breed dogs [34], whereas tracheal collapse is more frequently seen in small breed dogs [12,13]. This is a limitation, and caution should be taken when extrapolating our results to the general population. Further studies could help to determine more accurately the signalment associations and risk factors of dogs with these conditions.

The definition of paroxysmal coughing has been well established in human medicine [16], but it has not been widely described in veterinary medicine. In this manuscript, we referred to sudden onset and/or violent coughing episodes when using the term paroxysmal coughing. However, this is not a standardised definition and ultimately, due to the retrospective nature of this study, it was not possible to know how the clinicians in charge of each case interpreted paroxysmal coughing in detail. Further studies evaluating coughing characteristics in dogs could be considered to set a standardised interpretation that may help owners and veterinarians to identify this clinical sign, and allow better characterisation of the coughing.

Another limitation of our study is that the body condition score of the dogs was not recorded which could have been assessed along the body weight. This would have allowed us to further evaluate if obesity could be a risk factor, or if a low body condition score increased the likelihood of certain diagnoses. A dog with a neoplastic process or laryngeal paralysis with dysphagia and regurgitation could be underweight and analysing the weight alone as a risk factor could have led to the misinterpretation of some results.

As already mentioned, patients with advanced cardiac disease were often not clinically stable enough to undergo investigations to rule out a primary respiratory disease and therefore, could not be included in the study. This could have influenced our study population, so caution should be advised when extrapolating the results to the general population. Understating the role of cardiac disease in chronic coughing requires further investigation.

Rather than a limitation, a contextual aspect to bear in mind is that this study took place in England and at a referral centre, and caution should be taken before extrapolating any conclusion to the general population. The authors want to emphasise that the inclusion of information regarding the diagnostic investigations that were performed does not pretend to act as a guidance of whether a test is necessary to evaluate its clinical utility.

## 5. Conclusions

Airway collapse and chronic bronchitis were the most frequent diagnoses followed by neoplasia and infectious bronchopneumonia in this population of dogs with chronic cough. Signalment, coughing characteristics, and concomitant clinical signs increased the odds of certain diagnoses (Table 7 and Table 8).

The results of our study can be used as guidance when planning the investigations of a dog with chronic coughing. For example, age and/or the presence of inappetence, lethargy, haemoptysis, and/or weight loss would bring neoplasia higher on the list of differential diagnoses, whereas for laryngeal paralysis, which was also more likely in older animals, the presence of the aforementioned clinical signs was not a risk factor. Chronic coughing, regurgitation, and higher body weight should raise a suspicion of laryngeal paralysis. Chronic non-productive coughing, lower body weight, paroxysmal coughing, and exercise intolerance would be suggestive of airway collapse, particularly in certain breeds such as Chihuahua, Pomeranian, and Yorkshire Terrier. Productive cough and nasal discharge were more common in infectious bronchopneumonia. Younger age, higher body weight, and haemoptysis were suggestive of airway foreign body. The presence of chronic coughing and sneezing in a young patient would make eosinophilic lung disease more likely. Lastly, there were no predictive factors identified for chronic bronchitis.

All these findings are valuable for prioritising differential diagnoses and consequently increasing the efficacy of the diagnostic plan. This prioritisation becomes even more important in cases where financial constraints are present and also to help the optimisation of veterinary resources/time in general.

## Figures and Tables

**Table 1:** Summary of signalment and coughing characteristics for the dogs included in the study (*n* = 329).

Continuous Variables	Mean	Standard Deviation	Median	Range
Age, months	101	44.2	110	5–193
Body weight, kg	17.2	11.2	14.7	1.3–51.8
Cough duration at presentation, weeks	35.5	44.2	20	8–288
Discrete variables				
Sex	31 entire females (9.4%), 117 neutered females (35.6%) 48 entire males (14.6%), 133 neutered males (40.4%)			
Breed	Number of breeds = 82; percentage of pure breed dogs = 84.2% Percentages for the main breeds: 10.9% Labrador Retriever, 7.0% Yorkshire Terrier, 5.5% Cocker Spaniel, 5.2% Springer Spaniel, 3.0% Pomeranian, 3.0% Chihuahua, 2.7% Cavalier King Charles Spaniel, 2.4% Labradoodle, and 2.4% Jack Russell Terrier Percentages of dogs by size: toy (<5 kg) 12.8%, small (5–10 kg) 21.0%, medium (10–25 kg) 38.6%, large (25–40 kg) 24.9%, and giant (>40 kg) 2.7%			
Type of Coughing	85.1% non-productive coughing and 14.9% productive coughing 16.1% paroxysmal coughing			

**Table 2:** Co-occurrence of diagnosis for chronic coughing in the dogs included in the study (*n* = 329) *.

	Airway Collapse	Neoplasia	Chronic Bronchitis	Eosinophilic Lung Disease	Infectious Bronchopneumonia	Airway Foreign Body	Laryngeal Paralysis	Epiglottic Retroversion	Total Cases
Airway collapse	67.6	2.0	5.9	1.0	14.7	0.0	5.9	1.0	102
Neoplasia		90.3	0.0	0.0	1.6	0.0	3.2	0.0	62
Chronic bronchitis			65.0	0.0	17.5	0.0	10.0	0.0	80
Eosinophilic lung disease				86.8	10.5	0.0	0.0	0.0	38
Infectious. bronchopneumonia					27.8	9.3	3.7	1.9	54
Airway foreign body						66.7	0.0	0.0	15
Laryngeal paralysis							44.8	0.0	29
Epiglottic retroversion								66.7	6
Total cases	102	62	80	38	54	15	29	6	

* the ‘total cases’ columns represent the total number of dogs for each diagnosis. The remaining cells show the percentage of dogs that had only one diagnosis or more than one. The intensity of the colour indicates the intensity of the co-occurrence. The cells that are remarked in black show which values were statistically significant for Fisher’s exact test (*p* < 0.05).

**Table 3:** Differences in the duration of the coughing, age, and body weight of the dogs at presentation for the different diagnoses compared to the total population (*n* = 329).

	Duration of Coughing, Weeks		Age, Months		Body Weight, kg	
	Mean	*p* *	Mean	*p* *	Mean	*p* *
Total	35.5		101.0		17.2	
Airway collapse	42.8	0.007	107.3	0.180	10.8	<0.001
Chronic bronchitis	38.2	0.364	108.1	0.191	19.0	0.039
Neoplasia	25.9	0.004	124.7	<0.001	21.6	<0.001
Infectious bronchopneumonia	39.1	0.360	88.7	0.030	17.1	0.947
Eosinophilic lung disease	34.7	0.737	63.3	<0.001	18.3	0.239
Laryngeal paralysis	38.1	0.481	136.1	<0.001	28.3	<0.001
Airway foreign body	16.9	0.028	47.7	<0.001	24.9	0.005
Epiglottic retroversion	93.3	0.026	47.8	0.009	6.9	0.006
Aspiration pneumonia	41.5	0.455	99.5	0.931	12.7	0.511
Pulmonary fibrosis	83.0	0.716	128.0	0.212	10.2	0.277
Laryngitis	30.7	0.847	79.0	0.225	10.2	0.332
Other	21.8	0.567	72.6	0.021	10.8	0.009

* *p*-values for Wilcoxon–Mann–Whitney test.

**Table 4:** Chronic coughing in all those seen in a referral hospital from January 2012 to December 2021 (97 breeds and 41,856 cases).

Breed	Chronic Coughing Cases	Number of Patients	Percentage of Patients	*p*-Value *	Association with a Particular Diagnosis
Pomeranian	10	173	5.8	<0.001	Airway collapse
Yorkshire Terrier	23	603	3.8	<0.001	Airway collapse
Irish Terrier	2	53	3.8	0.016	
Shetland Sheepdog	3	115	2.6	0.030	
Toy Poodle	4	155	2.6	0.013	
Whippet	7	408	1.7	0.038	
Labradoodle	8	558	1.4	0.091	
Chihuahua	10	711	1.4	0.067	Airway collapse

* Fisher’s exact test.

**Table 5:** Diagnostic tests used more or less than average for each diagnosis of chronic coughing in 329 dogs. The percentage of patients for which the test was used within each diagnosis is indicated in parentheses.

Diagnostic	Diagnostic Tests Used More Than Average	Diagnostic Tests Used Less Than Average
Airway collapse	Upper airway assessment (78.2 **), BAL (84.2 **), thoracic radiographs (81.2 *), fluoroscopy (12.9 ***), echocardiography (29.7 ***), bronchoscopy (93.1 ***), and culture (79.2 *)	Thoracic ultrasound (1.0 **), CT (17.8 ***), FNA (5.0 ***), and histopathology (1.0 **)
Chronic Bronchitis	*Angiostrongylus vasorum* IDEXX Angio Detect Test^TM^ (47.5 ***), faecal analysis (18.8 **), upper airway assessment (77.5 *), BAL (100 ***), bronchoscopy (98.8 ***), PCR (45.0 ***), *Mycoplasma cynos* (38.8 ***), and culture (92.5 ***)	Thoracic ultrasound (1.3 *), fluoroscopy (0.0 **), FNA (2.5 ***), and histopathology (0.0 **)
Neoplasia	Coagulation times (12.9 *), thoracic ultrasound (32.3 ***), CT (71.0 ***), FNA (62.9 ***), and histopathology (33.9 ***)	*Angiostrongylus vasorum* IDEXX Angio Detect Test^TM^ (17.8 **), faecal analysis (1.6 *), upper airway assessment (17.7 ***), BAL (14.5 ***), thoracic radiographs (59.7 *), bronchoscopy (16.1 ***), PCR (1.6 ***), M. cynos (1.6 ***), and culture (14.5 ***)
Infectious bronchopneumonia	BAL (88.9 **), bronchoscopy (88.9 **), PCR (44.4 ***), *Mycoplasma cynos* (38.9 ***), *Bordetella bronchiseptica* (13.0 ***), and culture (94.4 ***)	FNA (5.6 *)
Eosinophilic lung disease	*Angiostrongylus vasorum* IDEXX Angio Detect Test^TM^ (57.9 ***), faecal analysis (21.1 *), upper airway assessment (81.6 *), BAL (100.0 ***), bronchoscopy (97.4 ***), *Angiostrongylus vasorum* PCR (10.5 **), *Crenosoma vulpis* PCR (7.9 **), and culture (97.4 ***)	FNA (2.6 *)
Laryngeal paralysis	Functional airway assessment (100.0 ***) and thoracic radiographs (89.7 *)	*Angiostrongylus vasorum* IDEXX Angio Detect Test^TM^ (13.8 *), CT (6.9 ***), and culture (48.3 *)
Airway foreign body		CBC (66.7*) and biochemistry (53.3 *)
Epiglottic retroversion	Upper airway assessment (100.0 †)	

Significance: † indicates *p* < 0.10, * indicates *p* < 0.05, ** indicates *p* < 0.01, and *** indicates *p* < 0.001.

**Table 6:** Univariate analysis of the association between sex, type of cough, and clinical signs with each diagnosis.

Variable (Frequency)	Airway Collapse (102)	Chronic Bronchitis (80)	Neoplasia (62)	Infectious Bronchopneumonia (54)	Eosinophilic Lung Disease (38)	Laryngeal Paralysis (29)	Airway Foreign Body (15)	Epiglottic Retroversion (6)
Total (329)	30.7	24.3	18.8	16.4	11.6	8.8	4.6	1.8
Sex								
Female (148)	36.5	21.6	21.0	18.2	10.8	6.1	2.0	1.3
Male (181)	26.0	26.5	17.1	14.9	12.2	11.1	6.6 †	2.2
Neutered								
Yes (250)	30.0	26.6	20.4	14.8	7.8	9.2	4.8	2.0
No (79)	32.9	23.6	13.9	21.5	12.8	7.6	3.8	1.27
Productive cough								
Yes (49)	4.1 ***	22.5	16.3	32.7 **	20.4 *	6.1	12.2 *	2.0
No (280)	35.4	24.6	19.3	13.6	10.0	9.3	3.21	1.8
Exercise intolerance (74)	46.0 **	16.2 †	8.1 **	23.0	6.8	14.9 †	2.7	2.7
Paroxysmal coughing (53)	64.2 ***	15.1	5.7 **	17.0	5.7	1.9 †	1.9	1.9
Lethargy (32)	25.0	25.0	28.1	25.0	0.0 *	3.1	0.0	0.0
Dyspnoea (29)	48.3 *	20.7	13.8	13.8	3.5	13.8	0.0	6.9 †
Tachypnoea (28)	25.0	21.4	28.6	7.1	3.6	17.9 †	7.1	0.0
Sneezing (27)	25.9	33.3	3.7 *	25.9	29.6 **	0.0	0.0	3.7
Nasal discharge (16)	0.0 **	43.8 †	12.5	43.8 **	12.5	0.0	0.0	0.0
Weight loss (14)	14.3	0.0 *	64.3 ***	14.3	0.0	0.0	7.1	0.0
Inappetence (19)	21.1	5.3 †	47.4 **	21.1	10.5	5.3	0.0	0.0
Haemoptysis (13)	0.0 *	0.0 *	69.2 ***	7.7	15.4	0.0	15.4	0.0
Vomiting (12)	0.0 *	8.33	41.7 *	16.7	8.33	8.3	0.0	8.3
Dysphagia (10)	20.0	20.0	10.0	20.0	0.0	20.0	0.0	30.0 ***
Fever (9)	22.2	22.2	22.2	33.3	0.0	0.0	22.2 †	0.0
Stridor (8)	37.5	0.0	0.0	37.5	37.5 †	0.0	12.5	0.0
Retching (8)	62.5 †	12.5	12.5	12.5	12.50	0.0	0.0	0.0
Stertor (7)	57.1	14.3	0.0	14.3	14.3	14.3	0.0	0.0
Bark change (6)	16.7	16.7	16.7	0.0	0.0	50.0 *	0.0	16.7
Regurgitation (6)	16.7	0.0	0.0	16.7	16.7	33.3†	0.0	33.3 **
Tracheal pinch (5)	60.0	0.0	0.0	20.0	40.0	0.0	0.0	0.0
Syncopal episodes (5)	60.0	20.0	0.0	40.0	20.0	20.0	0.0	0.0
Cyanosis (4)	50.0	0.0	0.0	0.0	50.0 †	25.0	0.0	0.0

The percentage of each diagnosis in the whole population of chronic coughing patients is shown in the first row. Cells show the percentage of dogs that presented each diagnosis when each clinical sign was present. Significance: † indicates *p* < 0.10, * indicates *p* < 0.05, ** indicates *p* < 0.01, and *** indicates *p* < 0.001. Clinical signs with a frequency lower than 4 (<1%; Ocular discharge and Epistaxis) were not analysed.

**Table 7:** Odds ratio for the risk factors found to be significant in the multivariable analysis for each diagnosis.

Diagnosis	Significant Predictors	Odds Ratio (95% Confidence Interval)	*p*-Value
Airway collapse	Body weight, kg	0.92 (0.90, 0.95)	<0.001
	Productive cough	0.07 (0.02, 0.29)	<0.001
	Paroxysmal coughing	4.9 (2.2, 11.0)	<0.001
	Exercise intolerance	3.2 (1.7, 6.3)	<0.001
Chronic bronchitis	No significant predictors		
Neoplasia	Age, months	1.025 (1.014, 1.036)	<0.001
	Body weight, kg	1.048 (1.018, 1.080)	0.002
	Lethargy	5.1 (1.5, 17.7)	0.010
	Exercise intolerance	0.12 (0.04, 0.38)	<0.001
	Haemoptysis	8.6 (1.9, 38.4)	0.005
	Weight Loss	4.0 (1.1, 15.3)	0.041
	Inappetence	6.5 (1.9, 22.1)	0.003
Infectious bronchopneumonia	Productive coughing	3.0 (1.5, 6.0)	0.002
	Nasal discharge	4.1 (1.4, 11.9)	0.009
Eosinophilic lung disease	Age, months	0.975 (0.967, 0.984)	<0.001
	Sneezing	3.2 (1.2, 8.5)	0.022
Laryngeal paralysis	Age, months	1.041 (1.022, 1.061)	<0.001
	Body weight, kg	1.105 (1.061, 1.150)	<0.001
	Regurgitation	48.5 (4.2, 554.8)	0.002
Airway foreign body	Age, months	0.96 (0.95, 0.98)	<0.001
	Body weight, kg	1.097 (1.037, 1.161)	0.001
	Haemoptysis	11.8 (1.8, 78.5)	0.010
Epiglottic retroversion	Age, months	0.969 (0.943, 0.994)	0.017
	Dysphagia	42.5 (4.7, 382.7)	<0.001
	Regurgitation	11.6 (1.2, 113.1)	0.035

**Table 8:** Summary of the main diagnoses identified in this population of dogs and significant predictors for each diagnosis.

Diagnosis	Significant Predictors
Airway collapse	lower body weight
	non-productive coughing paroxysmal coughing
	exercise intolerance
Chronic bronchitis	no significant predictors
Neoplasia	older dogs
	higher body weight
	lethargy
	haemoptysis
	weight loss
	inappetence
Infectious bronchopneumonina	productive coughing
	nasal discharge
Eosinophilic lung disease	younger dogs
	sneezing
Laryngeal paralysis	older dogs
	higher body weight
	regurgitation
Airway foreign body	younger dogs
	higher body weight
	haemoptysis
Epiglottic retroversion	younger dogs
	dysphagia
	regurgitation

## Data Availability

The data are contained within the article. The data presented in this study are available in the tables and figures in the article “Retrospective study of chronic coughing in dogs in a referral centre in the UK: 329 cases (2012–2021)”.

## Supplementary Materials

The following supporting information can be downloaded at https://www.mdpi.com/article/10.3390/ani15020254/s1. Table S1: Association analysis between diagnostic tests and diagnosis. The ‘total’ column indicates the percentage of dogs that had each diagnostic test. The numbers in the remaining columns indicate the difference in the percentage of dogs that underwent a diagnostic test within a diagnosis compared to the total. The red colour indicates that the test was more frequently performed for that particular diagnosis. The blue colour means that the test was less frequently performed for that particular diagnosis. The intensity of the colour indicates the intensity of the relationship. The cells that are remarked in black show which values were statistically significant (*p* < 0.05).

## Abbreviations

The following abbreviations are used in this manuscript:

OD	Odds ratio
CI	Confidence interval
CT	Computed tomography
PCR	Polymerase chain reaction
BAL	Bronchoalveolar lavage
FNA	Fine needle aspirate
