# Supplementary material for: Retrospective Study of Chronic Coughing in Dogs in a Referral Centre in the UK: 329 Cases (2012–2021)
Source: Animals (Basel). 2025 Jan 17;15(2):254. doi: 10.3390/ani15020254 (PMC11758286; doi:10.3390/ani15020254)
Supplement: Supplementary file 1 [file animals-15-00254-s001.zip › animals-3426906-supplementary.pdf]

Table S1: Association analysis between diagnostic tests and diagnosis. The 'total' column indicates the percentage of dogs that had each diagnostic test. The numbers in the remaining columns indicate the difference in the percentage of dogs that underwent a diagnostic test within a diagnosis compared to the total. The red colour indicates that the test was more frequently performed for that particular diagnosis. The blue colour means that the test was less frequently performed for that particular diagnosis. The intensity of the colour indicates the intensity of the relationship. The cells that are remarked in black show which values were statistically significant ($p < 0.05$).

	Total	Airway collapse (n = 102)	Chronic bronchitis (n = 80)	Neoplasia (n =62)	Infectious bronchopneumonia (n = 54)	Eosinophilic lung disease (n = 38)	Laryngeal paralysis (n = 29)	Airway foreign body (n = 15)	Epiglottic retroversion (n = 6)
Complete blood count	85.7	-5.5	4.3	4.6	5	-4.1	0.5	-19	-2.4
Biochemistry	81.5	-2.3	1	4	3.7	-5.2	4.7	-28.2	18.5
Coagulation times	6.7	-2.7	-4.2	6.2	2.6	-1.4	-6.7	-6.7	-6.7
IDEXX Angio Detect Test™	31.6	-6.8	15.9	-13.8	11	26.3	-17.8	-11.6	-31.6
Faecal analysis	8.8	-4.8	10	-7.2	-1.4	12.3	-1.9	-8.8	-8.8
Upper airway assessment	65.4	12.8	12.1	-47.7	3.1	16.2	34.6	1.3	34.6
BAL	72.3	11.9	27.7	-57.8	16.6	27.7	-6.8	-12.3	-5.6
Thoracic radiographs	72.6	8.6	2.4	-12.9	-2.2	-6.8	17.1	-5.9	-22.6
Thoracic ultrasound	7.6	-6.6	-6.3	24.7	-3.9	-7.6	-4.1	-0.9	9.1
CT	36.2	-18.4	-2.4	34.8	2.7	0.6	-29.3	23.8	-19.5
Fluoroscopy	5.6	7.3	-5.6	-4	0	-0.3	1.3	-5.6	-5.6
Echocardiography	16.7	13	-4.2	-7	-5.6	-6.2	-2.9	-10	0
Bronchoscopy	75.1	18	23.7	-59	13.8	22.3	-13	11.6	8.2
FNA	14.9	-9.9	-12.4	48	-9.3	-12.3	-11.4	-14.9	1.8
Histology	7.3	-6.3	-7.3	26.6	-3.6	-7.3	-7.3	-0.6	-7.3

PCR	24.9	-0.1	20.1	-23.3	19.5	6.7	-4.2	-11.6	-24.9
Mycoplasma cynos PCR	20.7	2.1	18.1	-19.1	18.2	-2.3	0	-7.4	-20.7
Bordetella bronchiseptica PCR	3.7	-1.7	2.6	-3.7	9.3	1.6	-0.2	3	-3.7
Angiostrongylus vasorum PCR	1.8	-1.8	-0.5	-1.8	0.1	8.7	-1.8	-1.8	-1.8
Crenosoma vulpis PCR	1.2	-1.2	0.1	-1.2	-1.2	6.7	-1.2	-1.2	-1.2
Coronavirus PCR	0.3	0.7	-0.3	-0.3	-0.3	-0.3	-0.3	-0.3	-0.3
Parainfluenza PCR virus	0.9	1.1	-0.9	-0.9	1	1.7	-0.9	-0.9	-0.9
Pneumocystis carinii PCR	0.3	-0.3	-0.3	-0.3	-0.3	-0.3	-0.3	-0.3	-0.3
Mycoplasma canis PCR	4.9	0.1	2.6	-4.9	0.7	-2.3	2	-4.9	-4.9
Viral panel (PCR)	1.5	-0.5	-0.2	-1.5	0.4	3.8	2	-1.5	-1.5
Culture (bacterial and/or fungal)	68.4	10.8	24.1	-53.9	26	29	-20.1	4.9	-18.4
